# Comparison between the acoustic fundamental frequency of the voice and the vibration frequency of the vocal folds analyzed by digital kymography

**DOI:** 10.1590/2317-1782/20232022173en

**Published:** 2023-10-27

**Authors:** Déborah de Oliveira Albino, Ualisson Nogueira do Nascimento, Elisa Meiti Ribeiro Lin Plec, Marco Aurélio Rocha Santos, Ana Cristina Côrtes Gama

**Affiliations:** 1 Departamento de Fonoaudiologia, Faculdade de Medicina, Universidade Federal de Minas Gerais - UFMG - Belo Horizonte (MG), Brasil.; 2 Programa de Pós-graduação em Ciências Fonoaudiológicas, Faculdade de Medicina, Universidade Federal de Minas Gerais - UFMG - Belo Horizonte (MG), Brasil.; 3 Hospital das Clínicas, Universidade Federal de Minas Gerais - UFMG - Belo Horizonte (MG), Brasil.

**Keywords:** Voice, Speech Acoustics, Kymography, Vocal Fold, Tonal Height Discrimination

## Abstract

**Purpose:**

To compare the frequency of vocal fold opening variation, analyzed by digital kymography, with the fundamental voice frequency obtained by acoustic analysis, in individuals without laryngeal alteration.

**Methods:**

Observational analytical cross-sectional study. The participants were forty-eight women and 38 men from 18 to 55 years of age. The evaluation was made by voice acoustic analysis, by the habitual emission of the vowel /a/ for 3 seconds, and days of the week, and digital kymography (DKG), by the habitual emission of the vowels /i/ and /ɛ/. The measurements analyzed were acoustic fundamental frequency (f0), extracted by the Computerized Speech Lab (CSL) program, and dominant frequency of the variation of right (R-freq) and left (L-freq) vocal fold opening, obtained through the KIPS image processing program. The mounting of the kymograms consisted in the manual demarcation of the region by vertical lines delimiting width and horizontal lines separating the posterior, middle and anterior thirds of the Rima glottidis. In the statistical analysis, the Anderson-Darling test was used to verify the normality of the sample. The ANOVA and Tukey tests were performed for the comparison of measurements between the groups. For the comparison of age between the groups, the Mann-Whitney test was used.

**Results:**

There are no differences between the values of the frequency measurement analyzed by digital kymography, with the acoustic fundamental frequency, in individuals without laryngeal alteration.

**Conclusion:**

The values of the dominant frequency of the vocal folds opening variation, as assessed by digital kymography, and the acoustic fundamental frequency of the voice are similar, allowing comparison between these measurements in the multidimensional evaluation of the voice, in individuals without laryngeal alteration.

## INTRODUCTION

Starting in the 15^th^ century, with the appearance of the Renaissance movement, science has sought means of understanding and explaining the mechanisms of voice production^(1)^. Numerous theories have been published that attempt such a feat, among them the muco-undulatory theory, the chaos theory and the aerodynamic myo-elastic theory^(1)^. Each one of them proposes a different model of how voice production works, considering the various aspects involved in phonation, such as the anatomical structures of the vocal tract and larynx, and their different possibilities of biomechanical movement; the physical events involved in the aerodynamics of breathing; and the acoustic result of the voice^(1,2)^. Since then, technology has advanced in the instrumentalization of researchers and professionals in the area, in order to substantiate theoretical data based on scientific knowledge.

Currently, there are a variety of tests and assessment techniques that contribute to the understanding of the biomechanics and physiology of the sounds produced by the vibration of the vocal folds, contributing to the accuracy of diagnoses and effectiveness of the rehabilitation process of dysphonia^(2)^. These exams provide auditory, visual and acoustic data, and can be divided into three pillars: visual image of the larynx, acoustic voice analysis, and auditory-perceptual analysis of voice quality^(3)^.

^.^Some imaging exams frequently described in the literature are: i) direct laryngoscopy, one of the pioneering techniques for visualizing the vocal tract, described for the first time in 1895, after obtaining an appropriate light source for its performance, and which until today, after numerous technological advances, is a widespread exam in all over the world^(4)^; ii) videolaryngostroboscopy, which has been widely used since electronic equipment for performing the technique emerged in 1960^(4)^. This exam creates the illusion of slow-motion vibration of the muco-undulatory movement of the vocal folds (VFs) by capturing about 30 images per second while a pulsed light is emitted^(5)^. The possibility of analyzing several laryngeal parameters related to the vibration pattern of the structures made this technique popular in research and, consequently, in vocal clinic^(4,5)^; ii) electroglottography, a non-invasive exam that detects the electrical activity of vibrations in the glottic area and is able to describe parameters such as the duration and relative pattern of contact of the VF cycle by cycle (4); iii) digital kymography, a more recent high-tech exam, which combined with high-speed recording, generates kymograms that allow quantitative analysis of parameters such as amplitude and frequency of each vocal fold and cycle by cycle, measuring asymmetries between them^(5,6)^.

On the other hand, the voice acoustic analysis is responsible for measuring the characteristics of the sound, and it happens by means of specific programs, developed to determine measures such as: i) fundamental frequency (f_0_), defined by the quantity of cycles of vibration of the VFs per second; ii) jitter and shimmer, two perturbation measures that indicate the variability of f_0_ (jitter) and sound wave amplitude (shimmer), both in the short term; iii) harmonic-noise proportion, which quantifies the noise generated by the air turbulence through the glottic structures, among other measures^(7,8)^.

The auditory-perceptual analysis is a subjective technique of vocal evaluation, in which the evaluator auditorily detects the characteristics of the individual's vocal pattern and can classify it in different ways^(3)^. This technique is considered the gold standard in vocal assessment and relating it to the acoustic analysis data and to the vibration pattern of VFs found in some imaging exams is what guarantees a multidimensional voice assessment^(3,9)^.

Owing to the variety of tests and analysis techniques available for studies in the area of voice, and due to the fact that each one has its own advantages and limitations, it has become common in literature and in vocal clinic the integration and correlation of data from auditory, visual and acoustic analysis of voice production^(5)^. Thus, the multidimensional evaluation of the voice becomes feasible, which is extremely important, because by relating different evaluative data, it enables a broader understanding of the vocal pattern of each individual^(1,9)^.

One study that used high-speed kymography and acoustic voice analysis to measure the immediate effect of sonorated tongue vibration exercises and basal sound, identified that tongue vibration exercise in women resulted in a significant decrease in jitter and closed phase time of kymography, while the video kymographic parameters of time of the opening and open phases of VFs increased. This result indicates less effort and higher quality in vocal production^(10)^.

In another research, when observing the correlation between the parameters of videokymography and acoustic analysis of voice, the authors described a typical phenomenon in Mongolian singers, the “Kargyraa”, in which the vestibular folds vibrated together with the VFs during emission, with complete closure, reduced frequency and different phase of the VFs, and were responsible for modulating the sound of the singing voice. The authors observed a correlation between the videokymography parameters and the presence of subharmonics in acoustic voice analysis^(11)^.

Another research correlated the results of voice acoustic measurements, perceptual-auditory analysis, and parameters of videokymography to explain the impact of vibration of the vestibular folds on vocal quality in usual and whispered emissions, concluded that the vestibular folds can vibrate without causing damage to vocal quality and this depends on factors such as voice frequency and regularity of vibration of this structure^(12)^.

A study compared the videokymographic open quotient and vocal intensity and concluded that the increase in the intensity is correlated with the reduction in the open quotient, in addition to the involuntary increase in f_0_^(13)^. Another study, when determining the relationship between different open quotients and intensity, frequency and phonation modes, observed strong correlation between videokymography open quotients and the parameters analyzed, except for f_0_^(14)^. In another study, upon realizing that the size of the glottis area is closely related to the variations of f0, the authors arrived at results that suggested that the combined analysis of digital videokymography (DKG) parameters and acoustic data of the voice is promising and can help in the identification of different types of vocal qualities^(15)^.

All the studies mentioned above^(10,11,12,13,14,15)^ show the importance of correlating data from different vocal and laryngeal evaluations for a better understanding of the aspects that involve vocal production.

It was not possible to identify any study that compared the measures related to the vibration frequency of VFs from digital kymography and acoustic analysis. Knowing that this data can contribute to the multidimensional evaluation of the voice, and consequently help in the process of vocal production analysis, this study aims to compare the frequency of the variation of vocal fold opening, analyzed by digital kymography, with the fundamental frequency of the voice, obtained through acoustic analysis, in individuals without laryngeal alteration.

The results of this research can contribute to a better understanding between the correlation of acoustic f_0_ and the number of glottic cycles obtained by the DKG, enabling the correlation between acoustic voice data and functional data of the VFs vibration velocity.

## METHODS

The present cross-sectional analytical observational study was approved by the Research Ethics Committee of Universidade Federal de Minas Gerais (UFMG) under process number 1,126,016. The convenience sample was made up of men and women, normal upon laryngeal examination, assessed using high-speed videolaryngoscopy (HSV), and aged between 18 and 55 years. All participants signed the Informed Consent Form.

Normal laryngeal examination was those who showed no lesions in the VFs with symmetry and periodicity of mucosal wave, and complete glottic closure. The presence of posterior triangular glottic chink in women was considered physiological^(16)^.

Exclusion criteria were laryngeal signs of gastroesophageal reflux, pregnancy, menstrual or premenstrual period, smoking, cervical surgeries, hormonal diseases, laryngeal diseases, self-reported upper airway infections, and the presence of an exacerbated nauseous reflex that were impediments to perform the exam.

The evaluation process consisted of two exams, namely the HSV, later analyzed through the DKG and the acoustic analysis of the voice. All assessments were conducted at the Functional Health Observatory in Speech-Language Pathology of the School of Medicine at the Universidade Federal de Minas Gerais (OSF/UFMG).

The laryngeal exam consisted of a HSV evaluation performed by two otorhinolaryngologists. Each examination consisted of 2000 images per second, taken with a rigid 70° laryngoscope with 300W xenon light (KayPentax®, Lincoln Park, New Jersey) with a model 9710 high-speed color video-laryngoscopy system. The image resolution used was 512 x 512 pixels with 8-bit RGB color mode. The records obtained through the habitual emission of the vowels /i/ and /ɛ/, were evaluated by selecting the most appropriate sequence of images. Larynges without benign changes and with complete glottal closure were considered normal. The exams were analyzed by the two otorhinolaryngologists, by consensus. Both had more than five years of experience in laryngology.

Ninety-eight laryngeal images were analyzed, by one of the researchers, and 12 were excluded due to low sharpness or corrupted file that made DKG analysis impossible, resulting in a final number of 86 subjects with DKG data and acoustic measurements.

The data evaluated referred to 86 participants, one group consisting of 48 women, and one group consisting of 38 men, all without laryngeal changes or with complete glottal closure. The group of women had a mean age of 26.8 years (18 to 55 years; SD=6.83) and the group of men a mean age of 26.4 years (18 to 44 years; SD=5.82), with no age difference between the groups (p=0.906).

For the acoustic analysis we used the Kay Pentax® Computerized Speech Lab (CSL), model 6103, Multi-Dimensional Voice Program (MDVP)15 module, installed in a Dell® Optiplex GX260 computer, with a DirectSound® professional sound card and a Shure® unidirectional condenser microphone. The participants were taken to an acoustically treated room and asked to stand with their feet slightly apart and their mouths 10 cm away from the microphone, which was placed on a pedestal. The recording was captured with the emission of the vowel /a/ in the usual manner, for 3 seconds, followed by the days of the week.

The measure analyzed was the fundamental frequency (f_0_), extracted automatically in the aforementioned program, which quantifies the average number of glottal cycles that happen per second throughout the emission time, in Hertz. The divergence of the collection material requested in the two tests is due to the fact that the vowels /i/ and /ɛ/ provide a higher position of the larynx during emission, making it easier to capture images by HSV^(17,18)^.

**Figure 1:**
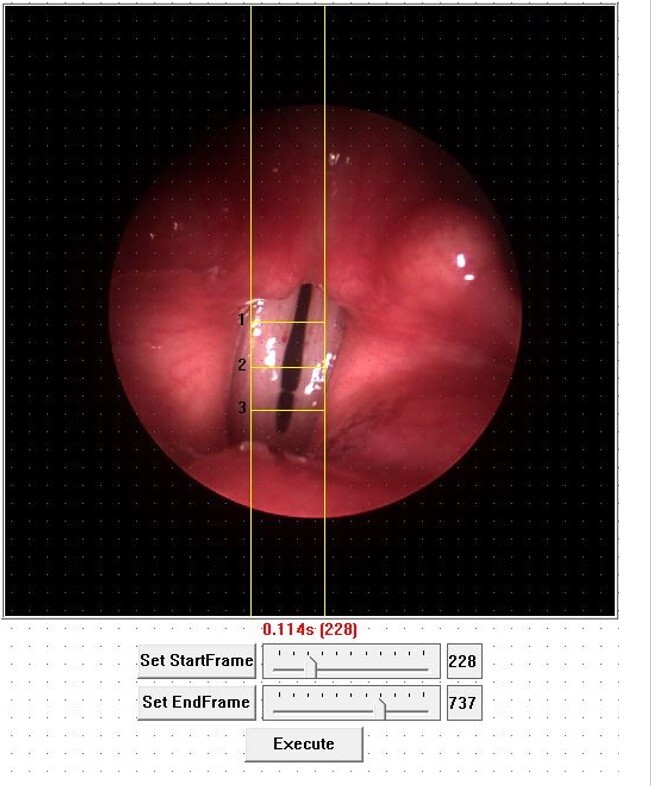
Manual demarcation of vertical and horizontal lines

Using the HSV laryngeal images we applied the image processing program called KIPS® (Kay's Image Processing Software), version 1.11, provided by KayPENTAX® for generation and analysis of digital kymography (DKG) parameters. The digital kymogram assembly process started with the manual demarcation of the region of the VFs to be analyzed in the images, consisting of two vertical lines, which delimit the width of the kymographic area, and 3 horizontal lines, representing the posterior thirds (line 1), demarcated below the vocal process of the arytenoids, middle (line 2) and anterior (line 3) in the Rima glottidis, and subsequently provided the frequency variation data (Figure 1).

**Figure 2:**
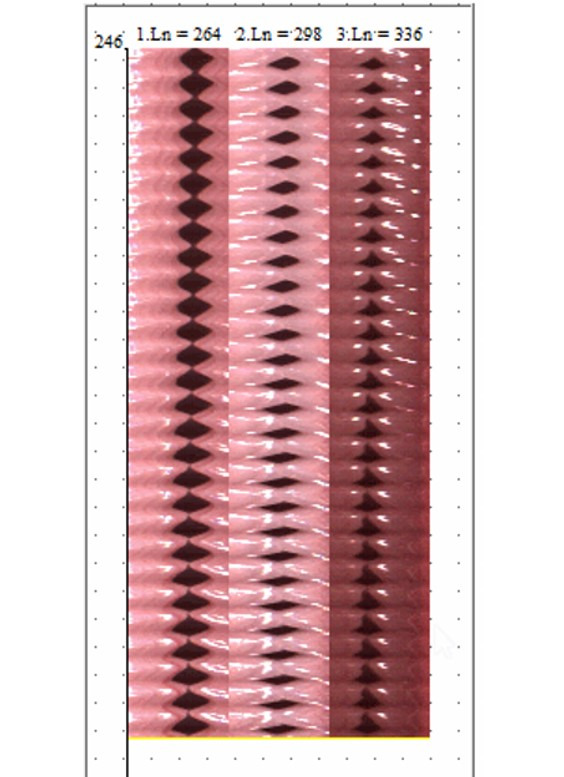
2-dimensional assembly of the kymogram

Subsequently, the beginning and end of the records were discarded and the program automatically performed the two-dimensional montage of the mucosal undulatory motion of the VFs (Figure 2).

**Figure 3:**
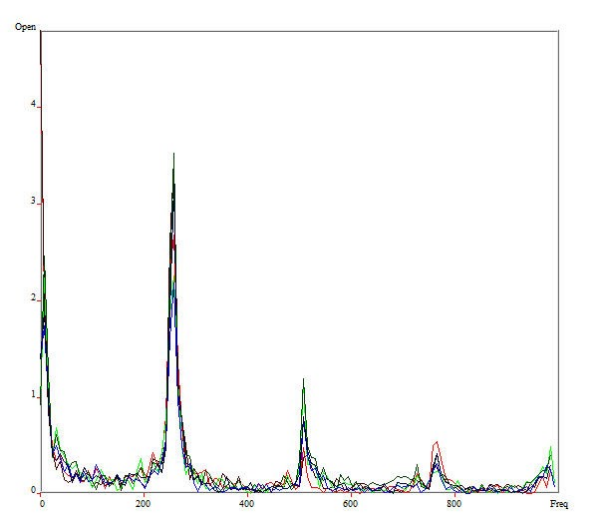
FFT plot with line overlap

**Figure 4:**
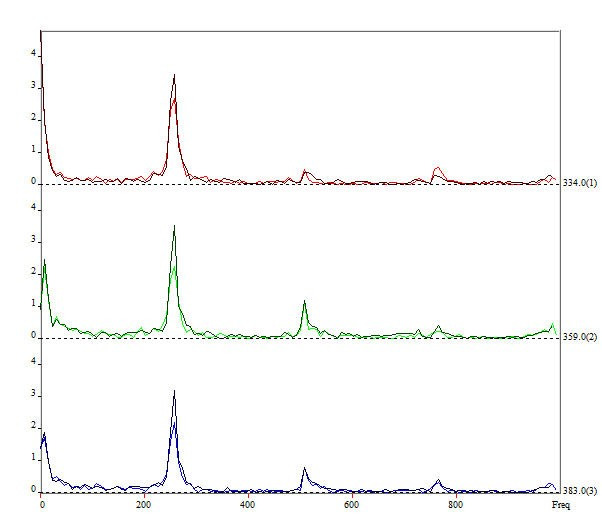
FFT plot with separate lines

Once the DKG graph was generated, the edge detection rectangle fitting tool and the edge detection tool were used to convert the images to grayscale, and the selection of the Rima glottidis for each line was finalized. Using this as a starting point, the program allows the analysis of the frequency variation of the lines, by means of the Fourier Transform (FFT), and can thus quantify the intensity changes of each pixel, image by image, and translate the information into graphs with frequency variation by time, so that the lines are overlapped (Figure 3) or separated (Figure 4). This tool provides in an objective way the cycle-by-cycle analysis of the vibration of the VFs.

**Caption:** Black lines = Represent the right vocal fold; Red, green and blue lines = Represent the left vocal fold in the posterior, middle and anterior thirds, respectively

KIPS® analyzed and quantified the data from the DKG graph and used in this research the parameters dominant frequency of the right vocal fold opening variation (R-freq) and dominant frequency of the left vocal fold opening variation (L-freq), both measured in pixels.

The demarcation of all laryngeal images was performed by one of the researchers, who showed an intra-rater agreement of 76% for the analysis of R-freq and 95% of L-freq. For analysis of agreement, 10 laryngeal images were duplicated.

The MINITAB program, version 17, was used for statistical analysis of the data. Initially, a descriptive analysis of the sample and the variables, with measures of central tendency and dispersion was performed. Afterwards, the Anderson-Darling test was applied to verify the normality of the variables. The ANOVA and Tukey tests were performed for multiple comparisons of the measurements between the groups. The Mann-Whitney non-parametric test was used to compare between age groups. To assess intra-rater agreement, the Intraclass Correlation Coefficient (ICC) was used in the PAST program (version 4.08). The evaluator showed excellent agreement^(19)^ for both parameters of the DKG.

## RESULTS

The results are evidenced in two tables, which show the comparison between the acoustic fundamental frequency and DKG frequency values of the right and left VFs, in rows 1, 2 and 3, in women (Table 1) and men (Table 2).

**Table 1:** Comparison of acoustic fundamental frequency and DKG frequency values of the right and left vocal folds in women without alteration

**WOMEN (n=48)**	Mean	Median	Standard Deviation	Lower limit	Upper limit	P-value
f_0_	214.81	211.72	30.06	206.08	223.54	
E1	216.96	218.75	31.67	207.76	226.16	
D1	216.96	218.75	31.59	207.79	226.13	
E2	215.95	218.75	31.93	206.68	225.22	0.982
D2	216.80	218.75	32.29	207.42	226.17	
E3	214.84	218.75	31.95	205.57	224.12	
D3	211.46	218.75	34.58	201.41	221.50	

**Caption:** f_0_ = Fundamental acoustic frequency; E1, E2, E3 = Dominant frequency of the opening variation of the left vocal fold (L-freq) in lines 1,2 and 3, by digital kymography (DKG); D1, D2, D3 = Dominant frequency of the opening variation of the right vocal fold (R-freq) in lines 1,2 and 3, by digital kymography (DKG); P-value = ANOVA test

**Table 2:** Comparison of acoustic fundamental frequency and DKG frequency values of the right and left vocal folds in men without alteration

**MEN (n=38)**	Mean	Median	Standard deviation	Lower limit	Upper limit	P-value
f_0_	129.82	122.00	31.44	119.48	140.15	
E1	136.92	125.00	35.30	125.32	148.53	
D1	136.51	125.00	34.78	125.08	147.94	
E2	136.51	125.00	34.78	125.08	147.94	0.960
D2	136.51	128.91	35.35	124.89	148.13	
E3	137.01	131.25	33.63	125.95	148.06	
D3	138.24	132.03	34.42	126.92	149.55	

**Caption:** f_0_ = Fundamental acoustic frequency; E1, E2, E3 = Dominant frequency of the opening variation of the left vocal fold (L-freq) in lines 1,2 and 3; D1, D2, D3 = Dominant frequency of the opening variation of the right vocal fold (R-freq) in lines 1,2 and 3; P-value = ANOVA test

There are no differences between the frequency values of the vocal fold opening variation, analyzed by DKG, with the f_0_ of the voice, obtained through acoustic analysis, in individuals without laryngeal alteration.

## DISCUSSION

The results of this research allow us to conclude that the acoustic f_0_ values ​​are correlated with the number of glottic cycles of the VFs, suggesting a correlation between the acoustic data of the number of sound waves, with the functional parameters of the VFs vibration velocity.

The f_0_ is defined as the number of oscillations of a wave in the interval of 1 second, given preferably in Hertz^(7)^. When applied to voice studies, this measurement corresponds to the number of cycles of the waves produced by the vibration of the VFs, and is studied and analyzed from 3 parameters: functional, auditory and acoustic^(3)^.

Regarding the functional field, the measure corresponds to the speed of vibration of the VFs, which combine the opening and closing movements in an almost cyclical manner (in normal voices) and result in voice production^(20)^. In auditory terms, frequency analysis corresponds to a subjective clinical practice, through the evaluator's impression of the patient's voice^(21)^. The auditory perception of frequency, with all its changes made by the vocal tract, is called pitch, and depends for its evaluation on psychosocial factors such as age and gender^(21,22)^. In the acoustic sphere, f0 is the lowest frequency detected in the voice signal, analyzed by means of specialized extraction algorithms^(23)^.

There are multiple ways of extracting or estimating the fundamental frequency, based on various mathematical models^(20,23)^. Each method is created based on a category of input domain, and the main ones are time domain and frequency domain^(20)^. In the time domain, there are event rate detection, autocorrelation, and phase space methods. Regarding the frequency domain there are the frequency component rate, filter-based, cepstral analysis, and multi-resolution methods^(20)^.

Peak rate is a time domain method that counts the number of wave peaks per second. In addition, the distance between the peaks reveals the wavelength, a measure inversely proportional to frequency^(20)^. Another widely used method is YIN, which consists of a combination of autocorrelation and cancellation techniques in the algorithm. Using the developed formulas, this method decreases the chance of subharmonic peak counting error^(20)^. An example of a frequency domain method is cepstral analysis, which takes into account regularly spaced partial frequencies and gives more linearity to the analysis by means of a logarithmic version of the Fourier Transform, which transforms the spectrum into a cepstrum^(20)^. Most algorithms have their own characteristics that can result in advantages or disadvantages in the estimation of the fundamental frequency of the voice, depending mainly on the type of sample^(20,22,23)^.

The correlation among functional, acoustic, and auditory aspects are of key importance for the multidimensional evaluation of the voice, which consists of the integration and interpretation of data obtained through phonoaudiological and otorhinolaryngological evaluations and the patient's self-perception of the vocal phenomenon^(1,9)^. Thus, by associating various methods of evaluation, this approach is able to detect losses not only physiological, as well as social and environmental^(1,9)^.

In this way, by stating that the measures of acoustic fundamental frequency and the dominant frequency of the opening variation of the VFs of DKG are equivalent, it contributes to the understanding of the correlation of acoustic and functional data, and consequently, for a more efficient and integral vocal evaluation. Such correlation is essential for a better functional understanding of the biomechanical process underlying the evaluated voice quality, which can bring important functional understandings in the case of dysphonia, helping in the decision-making of vocal techniques necessary for the desired functional rebalance in the speech-language pathology treatment process.

The findings of this research show mean acoustic f_0_ values for women of 214.81 Hz, and for men 129.82 Hz (Tables 1 and 2). These results are similar to what has been found in the literature, with mean values ranging from 194.09 Hz to 219.6 Hz for women and 118 Hz to 142 Hz for men^(6,24,25)^.

Regarding the digital kymographic frequency measurements, the mean values in the women's group ranged from 211.46 Hz to 216.96 Hz for left and right VFs (Table 1). The values were slightly lower than those found in the literature^(26,27)^ with women without laryngeal alteration, for lines 1 and 2, and similar in line 3.

In the men’s group, the mean values were 136.92 Hz to 138.24 Hz (Table 2) for the left and right VFs, respectively. These values are in agreement with the literature^(27,28)^.

It is important to point out that these tests are complementary, since the acoustic parameters are extracted from the vocal emission, and the DKG parameters from the laryngeal image. Despite being complementary, the results suggest that the aspects of vocal and laryngeal assessment are correlated, and that by acoustically analyzing the voice, the clinician can make inferences about the physiological aspects of the larynx. Considering the object of study of this research, the acoustic parameter of the voice related to f0 reflects the functional parameter of the number of glottal cycles.

It is worth mentioning as a limitation of the research the difference between the speech material used for the acoustic analysis (vowel /a/) and for the DKG (vowels /i/ and /ɛ/). In both acoustic f_0_ and DKG data collection, participants were monitored in relation to habitual emission, in frequency and intensity. However, the very nature of the HSV assessment, which requires tongue protrusion, prevents a habitual positioning of the vocal tract during the acquisition of images, and the emission of the vowels /i/ and /ɛ/ requires a slightly higher positioning of the larynx^(17)^. Both the protrusion of the tongue during the exam and the selected vowels are necessary for a better visualization of the VFs^(17)^. Such aspects may interfere in the measures analyzed; however, the research results allow us to conclude that these aspects did not interfere in the results.

There are few studies in the literature with data from digital kymography but comparing the dominant frequency data of the opening variation of the VFs with the acoustic fundamental frequency is a viable option, since these measures do not differ in men and women without vocal alteration.

## CONCLUSION

The comparison of the values of the dominant frequency of the VFs opening variation as evaluated by digital kymography, with the acoustic fundamental frequency of the voice show that they are similar, which allows a comparison between these measures in the multidimensional evaluation of the voice.
