# Supplementary material for: Comparison between the acoustic fundamental frequency of the voice and the vibration frequency of the vocal folds analyzed by digital kymography
Source: Codas. 2023 Oct 27;35(6):e20220173. doi: 10.1590/2317-1782/20232022173en (PMC10702710; doi:10.1590/2317-1782/20232022173en)
Supplement: Translation

# Translation: Comparação entre a frequência fundamental acústica da voz e a frequência de vibração das pregas vocais analisada pela videoquimografia digital

**DOI:** 10.1590/2317-1782/20232022173pt

**Authors:** Déborah de Oliveira Albino, Ualisson Nogueira do Nascimento, Elisa Meiti Ribeiro Lin Plec, Marco Aurélio Rocha Santos, Ana Cristina Côrtes Gama

**Affiliations:** 1 Departamento de Fonoaudiologia, Faculdade de Medicina, Universidade Federal de Minas Gerais – UFMG - Belo Horizonte (MG), Brasil.; 2 Programa de Pós-graduação em Ciências Fonoaudiológicas, Faculdade de Medicina, Universidade Federal de Minas Gerais – UFMG - Belo Horizonte (MG), Brasil.; 3 Hospital das Clínicas, Universidade Federal de Minas Gerais – UFMG - Belo Horizonte (MG), Brasil.

**Keywords:** Voz, Acústica da Fala, Quimografia, Prega Vocal, Discriminação da Altura Tonal

## Abstract

**Objetivo:**

Comparar a frequência da variação da abertura das pregas vocais, analisada pela videoquimografia digital, com a frequência fundamental da voz, obtida através da análise acústica, em indivíduos sem alteração laríngea.

**Método:**

Trata-se de um estudo observacional analítico transversal. Participaram 48 mulheres e 38 homens, de 18 a 55 anos. A avaliação foi composta por análise acústica da voz, obtida pela emissão habitual da vogal /a/ durante 3 segundos, e os dias da semana, e pela videoquimografia digital (DKG), obtida pela emissão habitual das vogais /i/ e /ɛ/. As medidas analisadas foram a frequência fundamental acústica (f_0_), extraída pelo programa Computerized Speech Lab (CSL), e a frequência dominante da variação de abertura da prega vocal direita (D-freq) e esquerda (E-freq), obtidas através do programa de processamento de imagens KIPS. A montagem dos quimogramas constou na demarcação manual da região, compostas por linhas verticais que delimitaram largura da prega vocal e linhas horizontais que marcaram os terços posterior, médio e anterior da rima glótica. Na análise estatística, o teste Anderson-Darling foi utilizado para verificar a normalidade da amostra. Os testes ANOVA e Tukey foram realizados para a comparação das medidas entre os grupos. Para a comparação da idade entre os grupos, foi utilizado o teste Mann-Whitney.

**Resultados:**

Não existem diferenças entre os valores da medida de frequência analisada pela videoquimografia digital, com a frequência fundamental acústica, em indivíduos sem alteração laríngea.

**Conclusão:**

Os valores da frequência dominante da variação de abertura das pregas vocais, avaliada pela videoquimografia digital, e a frequência fundamental acústica da voz são similares, permitindo uma comparação entre estas medidas na avaliação multidimensional da voz, em indivíduos sem alteração laríngea.

## INTRODUÇÃO

A partir do século XV, com o surgimento do movimento renascentista, a ciência busca meios de compreender e explicar os mecanismos de produção da voz^(1)^. Foram divulgadas inúmeras teorias que tentaram tal feito, dentre elas a teoria muco-ondulatória, a teoria do caos e a teoria mioelástica aerodinâmica, por exemplo^(1)^. Cada uma propõe um modelo próprio de explicação da produção vocal, levando em consideração os variados aspectos envolvidos na fonação, como as estruturas anatômicas do trato vocal e da laringe, e suas diferentes possibilidades de movimento biomecânico; os eventos físicos envolvidos na aerodinâmica da respiração; e o resultado acústico da voz^(1,2)^. Desde então, a tecnologia avança na instrumentalização dos pesquisadores e profissionais da área, a fim de fundamentar os dados teóricos com base nos conhecimentos científicos.

Atualmente, há uma diversidade de exames e técnicas de avaliação que contribuem para o entendimento da biomecânica e fisiologia dos sons produzidos pela vibração das pregas vocais, com vistas a contribuir com a precisão de diagnósticos e eficácia do processo de reabilitação das disfonias^(2)^. Esses exames, fornecem dados de naturezas auditiva, visual e acústica, e podem ser divididos em três pilares: os de imagem visual da laringe, a análise acústica da voz, e a análise perceptivo-auditiva da qualidade vocal^(3)^.

Alguns exames de imagem de laringe frequentemente descritos na literatura são: i) laringoscopia direta, uma das técnicas pioneiras de visualização do trato vocal, descrito pela primeira vez em 1895, após a obtenção de uma fonte de luz apropriada para sua realização, e que até hoje, após inúmeros avanços tecnológicos, é um exame difundido em todo o mundo^(4)^. ii) videolaringoestroboscopia, que foi amplamente utilizado a partir de 1960, quando os equipamentos eletrônicos para a realização da técnica surgiram^(4)^. Esse exame cria a ilusão de vibração em câmera lenta do movimento muco-ondulatório das pregas vocais (PPVV), ao capturar cerca de 30 imagens por segundo enquanto uma luz pulsátil é emitida^(5)^. A possibilidade de analisar vários parâmetros laríngeos relacionados ao padrão de vibração das estruturas o tornaram comum na pesquisa e, consequentemente, na clínica vocal^(4,5)^; iii) eletroglotografia, um exame não invasivo que detecta a atividade elétrica entre a área glótica, e que é capaz de descrever parâmetros como a duração e padrão relativo de contato das PPVV ciclo a ciclo^(5)^; iv) videoquimografia digital (DKG), um exame de alta tecnologia, mais recente, que combinado com a gravação em alta velocidade, gera quimogramas que permitem a análise quantitativa de parâmetros como amplitude e frequência de cada prega vocal ciclo a ciclo, e pode medir assimetrias entre elas^(5,6)^.

A análise acústica da voz é responsável por mensurar as características do som, e se dá por meio de programas específicos, desenvolvidos para determinar medidas como: i) frequência fundamental (f_0_), definida pela quantidade de ciclos de vibração das PPVV por segundo; ii) *jitter* e *shimmer,* duas medidas de perturbação que indicam a variabilidade da f_0_ *(jitter)* e da amplitude da onda sonora *(shimmer)*, ambas a curto prazo; iii) proporção harmônico-ruído, que quantifica o ruído gerado pelo turbilhonamento do ar através das estruturas glóticas, dentre outras medidas^(7,8)^.

Já a análise perceptivo-auditiva é uma técnica subjetiva de avaliação vocal, em que o avaliador detecta auditivamente as características do padrão vocal do indivíduo e pode classificá-lo de diferentes formas^(3)^. Essa técnica é considerada padrão-ouro na avaliação vocal e relacioná-la aos dados da análise acústica e ao padrão de vibração das PPVV encontrado em alguns exames de imagem é o que garante uma avaliação multidimensional da voz^(3,9)^.

Dada a variedade de exames e técnicas de análise disponíveis para os estudos na área de voz, e devido ao fato de que cada um possui suas vantagens e limitações próprias, se tornou comum na literatura e na clínica vocal a integração e correlação de dados das análises auditiva, visual e acústica da produção da voz^(5)^. Dessa forma, se torna viável a avaliação multidimensional da voz, que é de extrema importância, pois ao relacionar diferentes dados avaliativos, possibilita o entendimento mais amplo do padrão vocal de cada indivíduo^(1,9)^.

Uma pesquisa que utilizou a videoquimografia de alta velocidade e a análise acústica da voz para mensurar o efeito imediato dos exercícios de vibração sonorizada de língua e do som basal, identificou que o exercício de vibração de língua nas mulheres resultou na diminuição significativa do *jitter* e do tempo da fase fechada da videoquimografia, enquanto os parâmetros videoquimográficos de tempo das fases de abertura e aberta das PPVV aumentou. Esse resultado indica menor esforço e maior qualidade na produção vocal^(10)^.

Em outro estudo, ao observarem a correlação entre os parâmetros da videoquimografia e da análise acústica da voz, os autores descreveram um fenômeno típico nos cantores da Mongólia, o “Kargyraa”, em que as pregas vestibulares vibraram em conjunto com as PPVV durante a emissão, com fechamento completo, frequência reduzida e fase diferente das PPVV, e foram responsáveis por modular o som da voz cantada. Os autores observaram uma correlação entre os parâmetros da videoquimografia e a presença de subharmônicos na análise acústica da voz^(11)^.

Uma pesquisa que correlacionou os resultados das medidas acústicas da voz, da análise perceptivo-auditiva e de parâmetros da videoquimografia para explicar o impacto da vibração das pregas vestibulares na qualidade vocal nas emissões habitual e sussurrada, concluiu que as pregas vestibulares podem vibrar sem causar prejuízo à qualidade vocal e isso depende de fatores como a f_0_ da voz e a regularidade da vibração dessa estrutura^(12)^.

Um estudo comparou o quociente de abertura videoquimográfico e a intensidade vocal, e concluiu que o aumento da intensidade da emissão se correlaciona com a redução do quociente de abertura, além do aumento involuntário da f_0_^(13)^. Outro estudo, ao determinar a relação entre variados quocientes de abertura e intensidade, frequência e modos de fonação, observou fortes relações entre os quocientes de abertura da videoquimografia e os parâmetros analisados, exceto na f_0_^(14)^. Em outro trabalho, ao perceberem que o tamanho da área da glote tem relação íntima com as variações de f_0_, os autores chegaram a resultados que sugeriram que a análise combinada de parâmetros da DKG e dados acústicos da voz é promissora e pode auxiliar na identificação de diferentes tipos de qualidades vocais^(15)^.

Todos os estudos acima citados^(10,11,12,13,14,15)^ evidenciam a importância da correlação de dados de diferentes avaliações vocais e laríngeas para uma melhor compreensão dos aspectos que envolvem a produção vocal.

Não foi possível identificar nenhum estudo que comparasse as medidas relativas à frequência de vibração das PPVV da DKG e da análise acústica. Sabendo que esse dado pode contribuir com a avaliação multidimensional da voz, e consequentemente auxiliar no processo de análise da produção vocal, o presente estudo objetiva comparar a frequência da variação da abertura das PPVV, analisada pela DKG, com a f_0_ da voz, obtida através da análise acústica, em indivíduos sem alteração laríngea. Os resultados desta pesquisa podem contribuir para a melhor compreensão entre a correlação da f_0_ acústica e o número de ciclos glóticos obtidos pela DKG, possibilitando a correlação entre dados acústicos da voz e funcionais da velocidade de vibração das PPVV.

## MÉTODO

Trata-se de um estudo observacional analítico transversal aprovado pelo Comitê de Ética em Pesquisa sob parecer número 1.126.016. A amostra de conveniência foi composta por homens e mulheres com laringe normal, avaliada por meio da videolaringoscopia de alta velocidade (HSV), e com idade entre 18 e 55 anos. Todos os participantes assinaram o Termo de Consentimento Livre e Esclarecido. Considerou-se como exame laríngeo normal aqueles que apresentaram ausência de lesões nas PPVV, com simetria e periodicidade do movimento muco-ondulatório, e coaptação glótica completa. A presença de fenda triangular posterior nas mulheres foi considerada fisiológica^(16)^.

Os critérios de exclusão foram: sinais laríngeos de refluxo gastroesofágico, gravidez, período menstrual ou pré-menstrual, tabagismo, cirurgias na região cervical, doenças hormonais, doenças laríngeas, infecções autorreferidas de vias aéreas superiores e a presença de reflexo nauseoso exacerbado impedindo a realização do exame.

O processo de avaliação foi composto por dois exames, sendo eles a HSV, posteriormente analisada através da DKG e a análise acústica da voz. Todas as avaliações foram realizadas no Observatório de Saúde Funcional em Fonoaudiologia da Faculdade de Medicina da Universidade Federal de Minas Gerais (OSF/UFMG).

O exame de laringe constou de avaliação por HSV, realizada por dois médicos otorrinolaringologistas. Cada exame foi constituído de 2000 imagens por segundo, realizadas por um laringoscópio rígido de 70° com 300W de luz xênon (KayPentax ®, Lincoln Park, New Jersey) com sistema de HSV colorido modelo 9710. A resolução da imagem utilizada foi 512 x 512 pixels com modo de cor de 8 bit RGB. Os registros obtidos através da emissão habitual das vogais /i/ e /ɛ/, foram avaliados com seleção da sequência mais adequada de imagens. Os exames foram analisados pelos dois médicos otorrinolaringologistas, por consenso. Ambos apresentavam mais de cinco anos de experiência em laringologia.

Foram analisadas, por um dos pesquisadores, 98 imagens laríngeas, e 12 foram excluídas por baixa nitidez ou arquivo corrompido que impossibilitaram sua análise, resultando o número final de 86 sujeitos com dados de DKG e medidas acústicas.

Os dados avaliados se referem a 86 participantes, um grupo composto por 48 mulheres, e um composto por 38 homens. O grupo de mulheres apresentou idade média de 26,8 anos (18 a 55 anos; DP=6,83) e o grupo de homens idade média de 26,4 anos (18 a 44 anos; DP= 5,82), sem diferença de idade entre os grupos (p=0,906).

Para a análise acústica, foi utilizado o programa *Computerized Speech Lab* (CSL) da Kay Pentax®, modelo 6103, módulo *Multi-Dimensional Voice Program* (MDVP)15, instalado em um computador da marca Dell®, modelo Optiplex GX260, com placa de som profissional marca DirectSound® e microfone unidirecional, condensador, da marca Shure®. Os participantes foram encaminhados a uma sala acusticamente tratada, orientados a se posicionar de pé, com os pés sutilmente afastados, e com a boca a 10 cm de distância do microfone, apoiado sobre um pedestal. A gravação foi captada com a emissão da vogal /a/ de maneira habitual, durante 3 segundos, seguida dos dias da semana.

A medida analisada foi a frequência fundamental (f_0_), extraída de forma automática no programa supracitado, que quantifica a média das ondas sonoras por segundo ao longo do tempo da emissão, em Hertz. A divergência do material de coleta solicitado nos dois exames se dá pelo fato das vogais /i/ e /ɛ/ proporcionarem uma posição mais elevada da laringe durante a emissão, facilitando a captação de imagens pela HSV^(17,18)^.

A partir das imagens laríngeas da HSV, foi utilizado o programa de processamento de imagens denominado KIPS® (Kay´s Image Processing Software), versão 1.11, fornecido pela KayPENTAX® para geração e análise dos parâmetros da DKG. O processo de montagem dos videoquimogramas iniciou com a demarcação manual da região das PPVV a ser analisada nas imagens, composta por duas linhas verticais, que delimitam a largura da área quimográfica, e 3 linhas horizontais, que representam os terços posterior (linha 1), demarcado abaixo do processo vocal das aritenoides, médio (linha 2) e anterior (linha 3) na rima glótica, e posteriormente forneceram os dados de variação de frequência (Figura 1).

**Figura 1:** Demarcação manual das linhas verticais e horizontais

Em seguida, o início e o final dos registros foram descartados e o programa executou automaticamente a montagem bidimensional do movimento muco ondulatório das PPVV (Figura 2).

**Figura 2:** Montagem bidimensional do quimograma

**Legenda:** Representação da fase aberta (open) e da frequência fundamental (freq)

Com o gráfico da DKG gerado, a ferramenta de ajuste de retângulo de detecção de borda e a ferramenta de detecção de bordas foram utilizadas para converter as imagens para escala de cinza, e a seleção da rima glótica para cada linha foi finalizada. A partir disso, o programa permite a análise da variação de frequência das linhas, por meio das Transformadas de Fourier (FFT), e pode assim quantificar as alterações de intensidade de cada pixel, imagem por imagem, e traduzir as informações em gráficos com variação de frequência por tempo, de maneira em que as linhas fiquem sobrepostas (Figura 3) ou separadas (Figura 4). Essa ferramenta proporciona de forma objetiva a análise ciclo a ciclo da vibração das PPVV.

**Figura 3:** Gráfico da FFT com sobreposição das linhas

**Legenda:** Linhas pretas = Representam a prega vocal direita; Linhas vermelha, verde e azul = Representam a prega vocal esquerda nos terços posterior, médio e anterior, respectivamente. As frequências apresentadas são de 334Hz, 359Hz e 383Hz

**Figura 4:** Gráfico da FFT com linhas separadas

O KIPS® analisou e quantificou os dados do gráfico da DKG e utilizou-se nessa pesquisa os parâmetros Frequência dominante da variação de abertura da prega vocal direita (D-freq) e Frequência dominante da variação de abertura da prega vocal esquerda (E-freq), ambas medidas em pixels.

A demarcação de todas as imagens laríngeas foi realizada por um dos pesquisadores, que apresentou concordância intra-avaliador de 76% para a análise da D-freq e de 95% da E-freq. Para análise da concordância foram duplicadas 10 imagens laríngeas.

Para a análise estatística dos dados utilizou-se o programa MINITAB, versão 17. Inicialmente, uma análise descritiva da amostra e das variáveis, com medidas de tendência central e dispersão foi realizada. Em seguida, o teste Anderson-Darling foi aplicado para verificar a normalidade das variáveis. Os testes ANOVA e Tukey foram realizados para a comparação múltipla das medidas entre os grupos. Para comparação da idade entre os grupos foi utilizado o teste não paramétrico de Mann-Whitney. Para avaliação da concordância intra-avaliador foi utilizado o Coeficiente de Correlação Intraclasse (CCI) no programa PAST (versão 4.08). O avaliador apresentou concordância excelente^(19)^ para ambos os parâmetros da DKG.

## RESULTADOS

Os resultados estão evidenciados em duas tabelas, que mostram a comparação entre os valores de f_0_ acústica e frequência da DKG das PPVV direita (D) e esquerda (E), nas linhas 1, 2 e 3, em mulheres (Tabela 1) e homens (Tabela 2).

**Tabela 1:** Comparação entre os valores de frequência fundamental acústica e frequência da DKG das pregas vocais direita e esquerda em mulheres sem alteração

**MULHERES (n=48)**	Média	Mediana	Desvio-Padrão	Limite Inferior	Limite Superior	p-valor
f_0_	214,81	211,72	30,06	206,08	223,54	
E1	216,96	218,75	31,67	207,76	226,16	
D1	216,96	218,75	31,59	207,79	226,13	
E2	215,95	218,75	31,93	206,68	225,22	0,982
D2	216,80	218,75	32,29	207,42	226,17	
E3	214,84	218,75	31,95	205,57	224,12	
D3	211,46	218,75	34,58	201,41	221,50	

**Legenda:** f_0_ = Frequência fundamental acústica; E1, E2, E3 = Frequência dominante da variação de abertura da prega vocal esquerda nas linhas 1,2 e 3, pela videoquimografia digital (DKG); D1, D2, D3 = Frequência dominante da variação de abertura da prega vocal direita nas linhas 1,2 e 3, pela videoquimografia digital (DKG); p-valor = Teste ANOVA

**Tabela 2:** Comparação entre os valores de frequência fundamental acústica e frequência da DKG das pregas vocais direita e esquerda em homens sem alteração

**HOMENS (n=38)**	Média	Mediana	Desvio-Padrão	Limite Inferior	Limite Superior	p-valor
f_0_	129,82	122,00	31,44	119,48	140,15	
E1	136,92	125,00	35,30	125,32	148,53	
D1	136,51	125,00	34,78	125,08	147,94	
E2	136,51	125,00	34,78	125,08	147,94	0,960
D2	136,51	128,91	35,35	124,89	148,13	
E3	137,01	131,25	33,63	125,95	148,06	
D3	138,24	132,03	34,42	126,92	149,55	

**Legenda:** f_0_ = Frequência fundamental acústica; E1, E2, E3 = Frequência dominante da variação de abertura da prega vocal esquerda nas linhas 1,2 e 3; D1, D2, D3 = Frequência dominante da variação de abertura da prega vocal direita nas linhas 1,2 e 3; p-valor = Teste ANOVA

Não existem diferenças entre os valores de frequência da variação da abertura das pregas vocais, analisada pela DKG, com a f_0_ da voz, obtida através da análise acústica, em indivíduos sem alteração laríngea.

## DISCUSSÃO

Os resultados desta pesquisa permitem concluir que os valores da f_0_ acústica se correlacionam com o número de ciclos glóticos das PPVV, sugerindo a correlação entre os dados acústicos do número de ondas sonoras, com os parâmetros funcionais da velocidade de vibração das PPVV.

A f_0_ é definida como o número de oscilações de uma onda no intervalo de um segundo, dada preferencialmente em Hertz^(7)^. Nos estudos de voz, essa medida corresponde ao número de ciclos das ondas produzidas pela vibração das PPVV, e é estudada e analisada a partir de três parâmetros: funcional, auditivo e acústico^(3)^.

No campo funcional, a medida corresponde à velocidade de vibração das PPVV, que combinam os movimentos de abertura e fechamento de maneira quase cíclica (em vozes normais) e resultam na produção da voz^(20)^. Em termos auditivos, a análise da frequência corrobora para uma prática clínica subjetiva, através da impressão do avaliador sobre a voz do paciente^(21)^. A percepção auditiva da frequência, com todas as suas modificações realizadas pelo trato vocal, é denominada *pitch,* e depende de fatores psicossociais como idade e gênero para ser avaliado^(21,22)^. Já na esfera acústica, f_0_ é a frequência mais grave detectada no sinal de voz, analisada por meio de algoritmos especializados de extração^(23)^.

Existem múltiplas maneiras de extração/estimação da f_0_, com base em variados modelos matemáticos^(20,21)^. Cada método é criado com base em uma categoria de domínio de entrada, as principais sendo domínio do tempo e o domínio da frequência^(20)^. No âmbito do domínio do tempo, se destacam os métodos de detecção da taxa de tempo do evento, os de autocorrelação e os de espaço de fase. No domínio da frequência há os métodos de taxa de componentes de frequência, baseado em filtro, análise cepstral e multi-resolução^(20)^.

“Taxa de pico” (*Peak rate*) é um método do domínio do tempo que contabiliza o número de picos de onda por segundo. Além disso, a distância entre os picos revela o comprimento da onda, uma medida inversamente proporcional à frequência^(20)^. Outro método amplamente utilizado é o YIN, que consiste em uma combinação de técnicas de autocorrelação e cancelamento no algoritmo. Ao aplicar as fórmulas desenvolvidas, esse método diminui a chance de erro de contagem de picos de subharmônicos^(20)^. Um exemplo de método do domínio da frequência é a análise cepstral, que leva em conta as frequências parciais regularmente espaçadas e confere mais linearidade à análise, por meio de uma versão logarítmica da transformada de Fourier, que transforma o espectro em *cepstrum* ^(20)^. A maior parte dos algoritmos apresenta características próprias que podem resultar em vantagens ou desvantagens na estimação da f_0_ da voz, a depender principalmente do tipo de amostra^(20,22,23)^.

A correlação entre os aspectos funcionais, acústicos e auditivos são de suma importância para a avaliação multidimensional da voz, que consiste na integração e interpretação de dados obtidos através da avaliação fonoaudiológica, otorrinolaringológica e da auto-percepção do paciente acerca do fenômeno vocal^(1,9)^. Assim, ao associar variados métodos de avaliação, essa abordagem é capaz de detectar prejuízos não apenas fisiológicos, bem como sociais e ambientais^(1,9)^.

Dessa forma, ao afirmar que as medidas de f_0_ acústica e a frequência dominante da variação de abertura das PPVV, da DKG são equivalentes, contribui-se para o entendimento da correlação de dados acústicos e funcionais, e por consequência, para uma avaliação vocal mais eficiente e integral. Tal correlação é imprescindível para uma melhor compreensão funcional do processo biomecânico subjacente à qualidade vocal avaliada, o que pode trazer compreensões funcionais importantes no caso das disfonias, auxiliando na tomada de decisão de técnicas vocais necessárias para o reequilíbrio funcional desejado no processo do tratamento fonoaudiológico.

Os achados desta pesquisa mostram valores médios de f_0_ acústica de mulheres de 214,81 Hz, e de homens 129,82Hz (Tabelas 1 e 2). Tais resultados são semelhantes ao que foi encontrado na literatura, com valores médios variando de 194,09 Hz a 219,6 Hz para mulheres e 118 Hz a 142 Hz para os homens^(6,24,25)^.

Com relação às medidas de frequência videoquimográfica, os valores médios no grupo de mulheres variaram de 211,46 Hz a 216,96 Hz para as PPVV esquerda e direita (Tabela 1). Os valores foram ligeiramente menores do que os encontrados na literatura^(26,27)^ com mulheres sem alteração laríngea, para as linhas 1 e 2, e semelhantes na linha 3.

No grupo de homens, os valores médios foram de 136,92 Hz a 138,24 Hz (Tabela 2) para as PPVV esquerda e direita. Esses valores estão de acordo com a literatura^(27,28)^.

Tais resultados sugerem que os valores de f_0_ acústica e a frequência de vibração das PPVV, avaliada pela DKG não se diferem, o que permite uma comparação entre estas medidas na análise multidimensional da voz.

Importante salientar que estes exames são complementares, já que os parâmetros acústicos são extraídos da emissão vocal, e os parâmetros da DKG da imagem laríngea. Apesar de complementares, os resultados sugerem que os aspectos de avaliação vocal e laríngea são correlacionados, e que ao se analisar acusticamente a voz, o clínico pode fazer inferências sobre os aspectos fisiológicos da laringe. Considerando o objeto de estudo desta pesquisa, o parâmetro acústico da voz relacionado à f_0_ reflete o parâmetro funcional do número de ciclos glóticos.

Vale destacar como limitação da pesquisa a diferença entre o material de fala utilizado para a análise acústica (vogal /a/) e para a DKG (vogais /i/ e /ɛ/). Tanto na coleta dos dados da f_0_ acústica quanto da DKG, os participantes foram monitorados em relação a emissão habitual, em frequência e intensidade. Entretanto, a própria natureza da avaliação da HSV, que demanda protrusão da língua, impede um posicionamento habitual do trato vocal durante a obtenção das imagens, e a emissão das vogais /i/ e /ɛ/ exige um posicionamento levemente mais alto da laringe^(17)^. Tanto a protrusão da língua durante o exame, quanto das vogais selecionadas, são necessárias para uma melhor visualização das PPVV^(17)^. Tais aspectos podem interferir nas medidas analisadas, porém, os resultados da pesquisa permitem concluir que estes aspectos não interferiram nos resultados.

Existem poucas pesquisas na literatura com dados da DKG, mas comparar os dados de frequência dominante da variação de abertura das PPVV com a f_0_ acústica é uma opção viável, visto que são medidas que não se diferenciam em homens e mulheres sem alteração vocal.

## CONCLUSÃO

Os valores da frequência dominante da variação de abertura das pregas vocais, avaliada pela videoquimografia digital, com a frequência fundamental acústica da voz são similares, o que permite uma comparação entre estas medidas na avaliação multidimensional da voz.
